# Probabilistic Assessment of the Dynamic Viscosity of Self-Compacting Steel-Fiber Reinforced Concrete through a Micromechanical Model

**DOI:** 10.3390/ma15082763

**Published:** 2022-04-09

**Authors:** Ángel De La Rosa, Gonzalo Ruiz, Enrique Castillo, Rodrigo Moreno

**Affiliations:** 1 ETS de Ingenieros de Caminos, C. y P., Universidad de Castilla-La Mancha, Av. Camilo José Cela s/n, 13071 Ciudad Real, Spain; gonzalo.ruiz@uclm.es; 2 Real Academia de Ingeniería, Don Pedro 10, 28005 Madrid, Spain; castie@unican.es; 3 Instituto de Cerámica y Vidrio (CSIC), C. Kelsen 5, Campus de Cantoblanco, 28049 Madrid, Spain; rmoreno@icv.csic.es

**Keywords:** self-compacting steel-fiber reinforced concrete, dynamic viscosity, micromechanical constitutive model, deterministic and probabilistic models, Bayesian analysis

## Abstract

This article develops a probabilistic approach to a micromechanical model to calculate the dynamic viscosity in self-compacting steel-fiber reinforced concrete (SCSFRC), which implies a paradigm shift in the approach of the deterministic models used. It builds on a previous work by the authors in which Bayesian analysis is applied to rheological micromechanical models in cement paste, self-compacting mortar, and self-compacting concrete. As a consequence of the varied characteristics of the particles in these suspensions (in terms of materials, shapes, size distributions, etc.), as well as their random nature, it seems appropriate to study these systems with probabilistic models. The Bayesian analysis, thorough Markov Chain Monte Carlo and Gibbs Sampling methods, allows the conversion of parametric-deterministic models into parametric-probabilistic models, which results in enrichment in engineering and science. The incorporation of steel fibers requires a new term in the model to account for their effect on the dynamic viscosity of SCSFRC, and this new term is also treated here with the Bayesian approach. The paper uses an extensive collection of experimental data to obtain the probability density functions of the parameters for assessing the dynamic viscosity in SCSFRC. The results obtained with these parameters’ distributions are much better than those calculated with the theoretical values of the parameters, which indicates that Bayesian methods are appropriated to respond to questions in complex systems with complex models.

## 1. Introduction

Understanding the rheological behavior of cementitious suspensions is essential for new technological applications of concrete [1,2], such as pumping processes or digital manufacturing [3,4,5,6,7,8,9,10], as well as to carry out specific numerical simulations [11,12,13]. Advanced methods for the design of high-performance concrete [14,15,16,17] require knowing the values of their main rheological parameters. Particularly, the dynamic viscosity of this type of cementitious suspensions can be calculated from the experimental flow curve (shear stress–shear rate) using a Bingham-type linear fit model or estimated with the Krieger and Dougherty analytical equation [18] which correctly adjusts experimental rheological measurements carried out on cement pastes [19,20]. Besides, this analytical model is the foundation of advanced design methodologies for self-compacting concrete [14] and self-compacting steel-fiber reinforced concrete [15,17].

The Krieger and Dougherty equation, see Equation (1), consists of three parameters that have physical significance: The dynamic viscosity of the fluid phase, the maximum packing fraction, and the intrinsic viscosity of the particles (disperse phase). The dynamic viscosity of the fluid phase can be measured with greater or lower precision depending on the degree of complexity that it has in terms of being able to be considered in this phase, again, as a suspension [21]. The shape and the size distribution of the particles are the parameters on which the maximum packing fraction of the disperse solid phase, $\phi_{m}$, depends [19,22,23]. The intrinsic viscosity, [η], measures the individual effect of particles on viscosity [19,22]. It is a parameter closely related to the characteristics of the aggregates as well [24,25,26], i.e., the shape, the angularity, the roughness [27], and the circularity of the particles [24,26].

(1) $$\frac{\eta }{\eta_{0}}={\left(1\;-\;\frac{\phi }{\phi_{m}}\right)}^{-\left[\eta \right]\;\phi_{m}}$$

where

η: Suspension’s dynamic viscosity.
$\eta_{0}$: Fluid phase’s dynamic viscosity.
ϕ: Solid phase’s volume fraction.
$\phi_{m}$: Maximum packing fraction of particles.
[η]: Intrinsic viscosity of the system.

Self-compacting steel-fiber reinforced concrete (SCSFRC) is more complex than self-compacting concrete as a consequence of the inclusion of needle-shaped particles (steel fibers) which interact with granular and powder materials, giving rise to a more heterogeneous cementitious suspension. This polydisperse system of particles in suspension in a viscous homogeneous fluid phase makes it challenging to measure its rheological behaviour. Thus, it is necessary to use analytical or semi-empirical models that offer a good approximation of the rheological parameters of the suspension. If the Krieger and Dougherty equation allows estimating the dynamic viscosity of a cementitious suspension, such as cement paste or self-compacting mortar and concrete, other micromechanical models make it possible to predict the increase in dynamic viscosity produced by the addition of steel fiber into concrete [15,22,28,29].

The uncertainty associated with the variability of the rheological behavior in this type of cementitious suspension makes it interesting to convert this type of deterministic model to a model with random variables. We performed this Bayesian analysis of Equation (1) applied to cement paste, self-compacting mortar, and self-compacting concrete in a previous paper [21].

Bayesian statistics is an alternative to classical statistics since it allows defining the model parameters as random variables. In contrast, classical statistics would describe them with fixed values [30]. Bayesian statistics combine existing information about a problem and empirically observed data using probability guidelines, resulting in more reliable estimations and predictions [31]. Besides, Bayesian methodology allows obtaining large samples of the random variables (the parameters of the model) which can be considered as probability density functions instead of getting the point estimates of the parameters, which would be the object of classical statistics [30]. This fact supposes an enrichment of the models by offering unambiguous probabilistic information on the parameters of interest, which supposes a change of paradigm when proposing a model in engineering.

This article extends our work in [21] to SCSFRC, and its thesis consists in developing a methodology for the probabilistic assessment of the dynamic viscosity of SCSFRC through a micromechanical model. Thus, the purpose of the research is to apply a Bayesian methodology and enrich our model [15] by offering unambiguous probabilistic information on the parameters of interest. Moreover, we want to transform the cited deterministic model [15] into a probabilistic one with random variables. This topic falls at the core of rheology applied to SCSFRC, so it is of utmost importance for the technology of fiber concrete.

In this case, one more phase is added: Steel fibers. Based on the mix design methodology for SCSFRC developed by De La Rosa et al. [15], and with the experimental data obtained by Grünewald [32], a Bayesian analysis of the parameters of the constitutive models for estimating the dynamic viscosity of the suspension is done. We use the Krieger and Dougherty equation [18], and the Ghanbari et al. model [22] with the simplification proposed in [15]. Their parameters are considered here as random variables with their probability density functions, and not as unique values within confidence intervals. To our knowledge, this is the first time that this way of defining the parameters of a micromechanical model of phase suspension applied to the rheology of the SCSFRC has been considered.

The structure of the article is as follows. Firstly, we explain the essentials of Bayesian analysis, and how it facilitates the conversion of a deterministic model into a probabilistic one. Next, the paper gives details about the procedure and methodology. The following section describes the experimental data and the results. Finally, we draw the main conclusions from the investigation.

## 2. Probabilistic and Bayesian Analysis of a Micromechanical Constitutive Model to Calculate the Dynamic Viscosity in SCSFRC

Probabilistic network models are extensively used in engineering [33]. A key to implementing them is the definition of multivariate random variables, for which the Bayesian analysis provides a unique tool as it guarantees the existence of multivariate density functions.

The parameters $\phi_{m}$ and [η] of the equation of Krieger and Dougherty, Equation (1), which allows the prediction of the dynamic viscosity in cementitious suspensions, η, may be expressed in probabilistic terms as a consequence of the inherent random nature of the phenomenon. The same consequence can be drawn for the constitutive model of the fiber to be used. The idea arises from the fact that the parameters of Equation (1) can be treated as random variables, described by probability density functions, and not as a single value. Thus, the conversion of both models into probabilistic ones through the Bayesian analysis makes sense and is interesting for improving the assessment of the dynamic viscosity.

When we use frequentist statistics to calculate dynamic viscosity, it is considered a random variable of a parametric family. Thus, the problem is simplified to estimate the parameters of the equation. When Bayesian analysis is used, a set of parametric distribution families is taken into account, considering their parameters as random variables [34], thereby obtaining an extended family of mixtures that provides more freedom for the calculation process.

### 2.1. Sources of Randomness in Self-Compacting Steel-Fiber Reinforced Concrete

Self-compacting steel-fiber reinforced concrete may be understood as a system composed of several solid granular phases of one or various sizes (aggregates) with needle-shaped particles (steel fibers), all of them in a continuous phase, the cement paste [22]. The cement paste has an intrinsic random nature as a consequence of its colloidal behaviour and the interaction with superplasticizer molecules [34].

Aggregates are three-dimensional particles of different sizes, with irregular and random shapes, which influence the rheological properties of the cementitious suspensions of which they are a part. Their morphological characteristics are described by various geometric parameters related to dimensions, shape, angularity, surface roughness, etc. [35,36]. These parameters can be calculated through various techniques, such as digital image processing [37] or photogrammetry [38,39]. Considering the granular skeleton of the self-compacting concrete as a group of non-colloidal, rigid and polydisperse particles, the dynamic viscosity of the system can be estimated using Equation (1). The parameter $\phi_{m}$, which depends on the shape and the size distribution of particles [19,22,23], acquires a theoretical value of about 0.648 in a monodisperse rigid spherical system of particles (regardless of its size). $\phi_{m}$ reaches a theoretical value of 0.744 in polydisperse systems, where the space between particles can be filled efficiently [14,22]. Experimental data adjusted with Equation (1) indicate that $\phi_{m}$ shows a decreasing trend with decreasing maximum particle size, which is related to decreased polydispersity of the particles [27]. The intrinsic viscosity [η] is a measure of the individual effect of particles on viscosity [19,22]. It is a parameter closely related to the characteristics of the aggregates [24,25,26], namely their shape, angularity and roughness [27], as well as their circularity [24,26]. [η] is 2.5 for spherical and rigid particles [22], but when the particles deviate from this shape, [η] reaches different values [19,24,25,40,41]. Besides, the intrinsic viscosity appears to increase with decreasing maximum particle size, the cause of this phenomenon being unknown [27], which is a new source of randomness.

In SCSFRC the interactions between particles due to shear [22] have to be considered, together with the overall sizes and shapes (the high concentration of aggregates, mainly, and steel fibers). In Equation (1) [η] and $\phi_{m}$ depend on the shear rate, $\dot{\gamma }$, and the value of [η] $\phi_{m}$ is approximately constant if the assumption of rigid spheres is applied to aggregates ($\left[\eta \right]\;\phi_{m}\approx$ 1.9 [22] or 2 [42,43]). The shear rate energy is also another source of randomness in the suspension, as it happens in systems formed only by cement paste.

The volume fractions and the geometric shape of the fibers (even if they are in equivalent proportions of diluted systems) are another component of uncertainty to the system, since fibers interact with the aggregates, giving place to variations in the behavior of the whole suspension. Fibers are considered as slender rigid solids whose translation and rotation are conditioned by the resistance of the self-compacting viscous concrete matrix. In the micromechanical models available in the scientific literature to predict the increase in dynamic viscosity produced by the addition of steel fiber into concrete, the fiber content is limited to a maximum volume fraction of 2% (to consider the diluted concentration hypothesis) and a maximum aspect ratio equal to 85 (to fulfill the rigid solid hypothesis) [15,22].

Finally, these considerations must be taken into account when using the constitutive equation that calculates the increase in dynamic viscosity of self-compacting concrete due to the inclusion of the fiber.

### 2.2. Description of the Bayesian Methodology

The use of Bayesian methodology is well known [44,45,46] and has been widely described in a previous work [21]. In short, Bayesian methods allow to combine the information of the expert knowledge (which is subjective), given by the prior distribution, and the information of the sample knowledge (which is the observation of reality), through Bayes’ theorem, obtaining the posterior distribution (which is the combined one).

To apply the Bayesian methodology to a probabilistic model it is necessary to follow the next sequence [47]:

1. Choice of the likelihood family.
2. Choice of the prior distribution of the parameters:
  - By means of an imaginary sample (consulting an expert to provide a virtual sample representative of the prior knowledge).
  - Through previous non-updated information (consulting the expert).
  - Through our experimental data.
3. Obtaining data from the sample.
4. Calculation of the posterior distribution.
5. Through the combination of the posterior with the likelihood, the predictive distribution is obtained, which is the one we used.

Compared to frequentist statistics, Bayesian statistics have advantages such as obtaining better parameter estimations with small sample sizes, easy interpretation of the results when calculating the probabilities of the parameters, the introduction of measures of uncertainty, missing data and levels of variability [48].

### 2.3. Proposal of the Probabilistic Model and Bayesian Analysis of the Constitutive Model to Calculate the Dynamic Viscosity in SCSFRC

The purpose of this research is to convert the model to calculate the dynamic viscosity of SCSFRC into a parametric model using Bayesian analysis. The procedure considers SCSFRC as a heterogeneous material composed of fibers in suspension in a more or less homogeneous granular fluid, which is the self-compacting matrix.

It is important to have good prior information, acquired using the experimental data or through expert consultation (i.e., scientific literature). It is very important to discern the quality of the information, especially if there is not enough data [47]. The Bayesian model to be created (its network) will consider the randomness of the dynamic viscosity mean value, and also the variability of the parameters. Proceeding this way, the parametric-deterministic model can be converted into a parametric-probabilistic model through the open-source software OpenBUGS [49]. This software incorporates a Bayesian inference program using the Markov Chain Monte Carlo method (MCMC) and the Gibbs Sampling methodology, a particular case of simulation algorithm of a Markov Chain. The software creates an acyclic graph with the hierarchical dependence structure of variables and parameters, and the posterior probability density functions of the parameters, together with the statistical values of the probabilistic model.

#### Self-Compacting Steel-Fiber Reinforced Concrete Suspensions

Self-compacting steel-fiber reinforced concrete can be considered as a multi-phase suspension composed of a heterogeneous phase (self-compacting concrete matrix) and steel fibers in suspension. Equation (1) includes the solid phases (powder, fine and coarse aggregate), and allows calculating the increase of the dynamic viscosity through Equation (2) [14]:

(2) $$\eta^{\circ }={\left(1\;-\;\frac{\phi_{\mathrm{fa}}}{\phi_{m\;\mathrm{fa}}}\right)}^{-{\left[\eta \right]}_{\mathrm{fa}}\;\phi_{m\;\mathrm{fa}}}\;{\left(1\;-\;\frac{\phi_{\mathrm{FA}}}{\phi_{m\;\mathrm{FA}}}\right)}^{-{\left[\eta \right]}_{\mathrm{FA}}\;\phi_{m\;\mathrm{FA}}}\;{\left(1\;-\;\frac{\phi_{\mathrm{CA}}}{\phi_{m\;\mathrm{CA}}}\right)}^{-{\left[\eta \right]}_{\mathrm{CA}}\;\phi_{m\;\mathrm{CA}}}$$

where

$\eta^{\circ }=\frac{\eta_{\mathrm{SCC}}}{\eta_{p}}$: Self-compacting concrete dimensionless viscosity.
$\eta_{\mathrm{SCC}}$: Self-compacting concrete dynamic viscosity.
$\eta_{p}$: Cement paste dynamic viscosity.
$\phi_{\mathrm{fa}}$: Volume fraction of the powder phase.
$\phi_{m\;\mathrm{fa}}$: Particles’ maximum packing fraction of the powder phase.
${\left[\eta \right]}_{\mathrm{fa}}$: Intrinsic viscosity taking into account the powder phase.
$\phi_{\mathrm{FA}}$: Volume fraction of the fine aggregate phase.
$\phi_{m\;\mathrm{FA}}$: Maximum packing fraction of the fine aggregate phase.
${\left[\eta \right]}_{\mathrm{FA}}$: Intrinsic viscosity of the fine aggregate phase.
$\phi_{\mathrm{CA}}$: Volume fraction of the coarse aggregate phase.
$\phi_{m\;\mathrm{CA}}$: Maximum packing fraction of the coarse aggregate phase.
${\left[\eta \right]}_{\mathrm{CA}}$: Intrinsic viscosity of the coarse aggregate phase.

In Equation (2), the parameters are treated as random variables which follow a probability density function of uniform type, within a range of maximum and minimum values. This uninformative priors have been chosen in order to let the data make the adequate corrections. These corrections can be seen in the posteriors when they separate from the uniform trend. We must note that when dependence exists, relatively small sizes are sufficient to produce large changes in the posteriors, which justifies the selected uninformative priors.

Equation (2) calculates the mean value of the dynamic viscosity of SCC, which is assumed to follow a normal probability density function, where the mean value is $\mu^{\circ }$, and the standard deviation value is σ. $\epsilon^{\circ }$ is the residual value, which follows a normal family; moreover, $\epsilon^{\circ }$ includes a uniform function of density.

The syntax of the extended model of the Krieger and Dougherty equation in a statistical format is:

(3) $$\begin{array}{ccc} \eta^{\circ } & ∼ & N\;[\mu^{\circ },\nu ] \end{array}$$

(4) $$\begin{array}{ccc} \mu^{\circ } & = & {\left(1\;-\;\frac{\phi_{\mathrm{fa}}}{\phi_{m\;\mathrm{fa}}}\right)}^{-{\left[\eta \right]}_{\mathrm{fa}}\;\phi_{m\;\mathrm{fa}}}\;{\left(1\;-\;\frac{\phi_{\mathrm{FA}}}{\phi_{m\;\mathrm{FA}}}\right)}^{-{\left[\eta \right]}_{\mathrm{FA}}\;\phi_{m\;\mathrm{FA}}}\;{\left(1\;-\;\frac{\phi_{\mathrm{CA}}}{\phi_{m\;\mathrm{CA}}}\right)}^{-{\left[\eta \right]}_{\mathrm{CA}}\;\phi_{m\;\mathrm{CA}}} \end{array}$$

(5) $$\begin{array}{ccc} \phi_{m\;\mathrm{fa}} & ∼ & U\;[\phi_{m\;\mathrm{fa}\;\min},\;\phi_{m\;\mathrm{fa}\;\max}] \end{array}$$

(6) $$\begin{array}{ccc} \phi_{m\;\mathrm{FA}} & ∼ & U\;[\phi_{m\;\mathrm{FA}\;\min},\;\phi_{m\;\mathrm{FA}\;\max}] \end{array}$$

(7) $$\begin{array}{ccc} \phi_{m\;CA} & ∼ & U\;[\phi_{m\;CA\;\min},\;\phi_{m\;CA\;\max}] \end{array}$$

(8) $$\begin{array}{ccc} {\left[\eta \right]}_{\mathrm{fa}} & ∼ & U\;\left[{\left[\eta \right]}_{\mathrm{fa}\;\min},\;{\left[\eta \right]}_{\mathrm{fa}\;\max}\right] \end{array}$$

(9) $$\begin{array}{ccc} {\left[\eta \right]}_{\mathrm{FA}} & ∼ & U\;\left[{\left[\eta \right]}_{\mathrm{FA}\;\min},\;{\left[\eta \right]}_{\mathrm{FA}\;\max}\right] \end{array}$$

(10) $$\begin{array}{ccc} {\left[\eta \right]}_{\mathrm{CA}} & ∼ & U\;\left[{\left[\eta \right]}_{\mathrm{CA}\;\min},\;{\left[\eta \right]}_{\mathrm{CA}\;\max}\right] \end{array}$$

(11) $$\begin{array}{ccc} \sigma & ∼ & U\;[\sigma_{\min},\;\sigma_{\max}] \end{array}$$

The incorporation of the steel fiber is taken into account by means of the model proposed by De La Rosa et al. (Equation (12)) to design self-compacting steel-fiber reinforced concrete [15]:

(12) $$\eta_{SCSFRC}=\eta_{p}{\left(1\;-\;\frac{\phi_{\mathrm{fa}}}{\phi_{m\;\mathrm{fa}}}\right)}^{-{\left[\eta \right]}_{\mathrm{fa}}\;\phi_{m\;\mathrm{fa}}}\;{\left(1\;-\;\frac{\phi_{\mathrm{FA}}}{\phi_{m\;\mathrm{FA}}}\right)}^{-{\left[\eta \right]}_{\mathrm{FA}}\;\phi_{m\;\mathrm{FA}}}\;{\left(1\;-\;\frac{\phi_{\mathrm{CA}}}{\phi_{m\;\mathrm{CA}}}\right)}^{-{\left[\eta \right]}_{\mathrm{CA}}\;\phi_{m\;\mathrm{CA}}}\left(1+\frac{\phi_{f}}{\phi_{\lambda }}\right)$$

where

(13) $$\phi_{\lambda }=\frac{3\;\ln(2\lambda )}{\pi \;\lambda^{2}}$$

(14) $$\eta_{SCSFRC}=\eta_{SCC}\;\left(1+\frac{\phi_{f}}{\phi_{\lambda }}\right)$$

In Equation (13), λ is the aspect ratio of the steel fiber. It is obtained from the simplification of the model of Ghanbari et al. [22]. The only factor that can be parametrized is the number 3 which appears in both equations (the parameterization of this value will be carried out in Section 4).

Thus, the syntax of the model of De La Rosa et al. (Equation (12)) [15] in a statistical format is:

(15) $$\begin{array}{ccc} \eta^{\circ } & ∼ & N\;[\mu^{\circ },\nu ] \end{array}$$

(16) $$\begin{array}{ccc} \mu^{\circ } & = & {\left(1\;-\;\frac{\phi_{\mathrm{fa}}}{\phi_{m\;\mathrm{fa}}}\right)}^{-{\left[\eta \right]}_{\mathrm{fa}}\;\phi_{m\;\mathrm{fa}}}\;{\left(1\;-\;\frac{\phi_{\mathrm{FA}}}{\phi_{m\;\mathrm{FA}}}\right)}^{-{\left[\eta \right]}_{\mathrm{FA}}\;\phi_{m\;\mathrm{FA}}}\;{\left(1\;-\;\frac{\phi_{\mathrm{CA}}}{\phi_{m\;\mathrm{CA}}}\right)}^{-{\left[\eta \right]}_{\mathrm{CA}}\;\phi_{m\;\mathrm{CA}}}\;\left(1+\frac{\phi_{f}}{\phi_{\lambda }}\right) \end{array}$$

and Equations (6) to (12).

## 3. Materials and Methods

A set of 56 self-compacting steel-fiber reinforced concretes (SCSFRCs) from [32] has been analyzed. The granular skeleton that SCSFRC is composed of rounded fine aggregate (0.125–4 mm) and rounded coarse aggregate (4–8 mm and 4–16 mm). The steel fibers are included in a $\phi_{f}$ range of 0.003 to 0.02, and their λ value is between 46.3 and 85.7. Rheological measurements of self-compacting steel-fiber reinforced concretes were done using a coaxial cylindrical viscometer (BML–Viscometer). The procedure to calculate the rheological parameters was the following: The rotation velocity of the outer cylinder of the viscometer was increased up to its maximum value and, once it was reached, the velocity was decreased [32].

In Ghanbari et al. [22], the dynamic viscosity of the cement pastes was calculated from data from the scientific literature on their composition [14,22,50,51] according to Ghanbari et al. [22]. In Table 1 and Table 2, the composition of each base SCC, $\eta_{SCC}$, $\eta_{p}$, λ, $\phi_{f}$ and $\eta_{SCSFRC}$ are included. The self-compacting steel-fiber reinforced concretes studied are derived from nine self-compacting matrices elaborated by Grünewald (Table 1) [32]. Table 2 shows the values of λ, $\phi_{f}$, and $\eta_{SCSFRC}$ of the combinations of concretes developed in [32].

In the Bayesian analysis, a total of 11,000 iterations in every model were done, through OpenBUGS, to obtain the samples of the variables (parameters of the deterministic models) that are considered as their density functions.

The model analyzed is the one proposed by De La Rosa et al. to design SCSFRC [15], see Equation (12). Function $\phi_{\lambda }$, Equation (13), depends on the π number and the aspect ratio of the steel fibers. Thus, the factor that can be parametrized is the number 3 (=δ, i.e., the numerator of Equation (13)). It must be taken into account that the rest of the parameters of the model ($\phi_{\mathrm{mj}}$, ${\left[\eta \right]}_{j}$), corresponding to Equation (2), also have been subjected to Bayesian analysis to find their density functions.

## 4. Results and Discussion

### 4.1. Bayesian Analysis Model in Self-Compacting Steel-Fiber Reinforced Concrete

Firstly, it has been analyzed if the δ parameter approaches 3. For this purpose, we use the experimental data of dynamic viscosity measured by Grünewald [32] in SCC, $\eta_{SCC}$ and SCSFRC, $\eta_{SCSFRC}$. The model and the parameter definition domains for the Bayesian analysis, according to Equation (17), are:

(17) $$\frac{\eta_{SCSFRC}}{\eta_{SCC}}=\left(1+\frac{\phi_{f}}{\phi_{\lambda }}\right)$$

(18) $$\begin{array}{ccc} \eta^{⋄} & ∼ & N\;[\mu^{⋄},\nu ] \end{array}$$

(19) $$\begin{array}{ccc} \mu^{⋄} & = & \left(1+\frac{\pi \;\lambda^{2}\;\phi_{f}}{\delta \;\ln(2\lambda )}\right) \end{array}$$

(20) $$\begin{array}{ccc} \delta & ∼ & U\;[0,\;50] \end{array}$$

(21) $$\begin{array}{ccc} \sigma & ∼ & U\;[0,\;400] \end{array}$$

where

$\eta^{⋄}=\frac{\eta_{\mathrm{SCSFRC}}}{\eta_{SCC}}$: Non-dimensional viscosity of self-compacting steel-fiber reinforce concrete.
$\eta_{\mathrm{SCSFRC}}$: Self-compacting steel-fiber concrete dimensionless viscosity.
$\eta_{SCC}$: Self-compacting concrete dynamic viscosity.
$\phi_{f}$: Steel-fiber volume fraction.
λ: Steel-fiber aspect ratio.
δ: Parameter of the system when adding the steel fiber.

**Figure 1:**
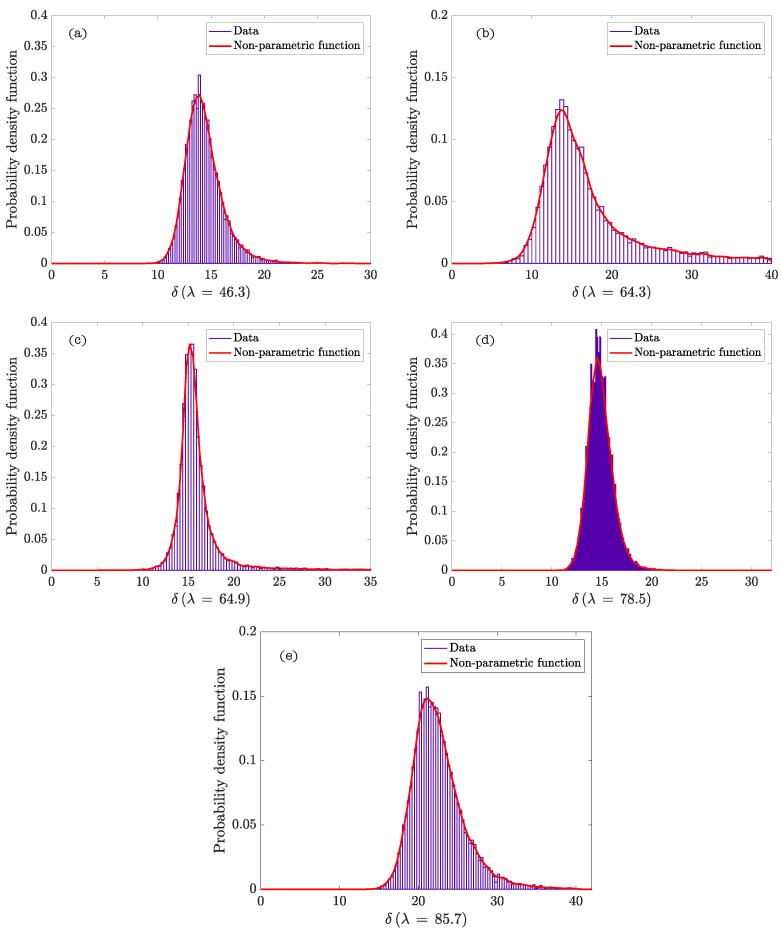
Probability density functions of δ for different values of λ (λ = 46.3 (**a**), 64.3 (**b**), 64.9 (**c**), 78.5 (**d**), and 85.7 (**e**)).

The upper value of the parameter δ (δ = 50) is selected to obtain a wide range of calculations. Table 3 contains the statistics values of δ once the analysis of the model has been done. Figure 1 represents the non-parametric density functions of the parameter δ calculated with Equation (17) for different values of λ.

At this point, we have to keep in mind that the main objective is to evaluate the feasibility of Equation (12) [15] to design SCSFRC. The material of the powder phase used in the experimental investigation of Grünewald [32] is fly ash. Two uniform random variables for ${\left[\eta \right]}_{\mathrm{fa}}$ are considered as priors in order to analyze the model. The first, ${\left[\eta \right]}_{fa}\;∼\;U\;\left[4.30,\;6.80\right]$, is obtained for cement pastes [21]. The second arises due to the sphericity of the fine particles of fly ash, which implies that the minimum value of ${\left[\eta \right]}_{\mathrm{fa}}$ must be reduced from 4.30 to 2.50. Therefore, the widest range will be used in the analysis of the parameter ${\left[\eta \right]}_{fa}$, i.e., ${\left[\eta \right]}_{fa}\;∼\;U\;\left[2.50,\;6.80\right]$.

The parameters of Equations (15) and (16) of the model [15] in a statistical format are defined in the following values:

$$\begin{array}{ccc} \phi_{m\;\mathrm{fa}} & ∼ & U\;[0.550,\;0.830]\\ \phi_{m\;\mathrm{FA}} & ∼ & U\;[0.550,\;0.717]\\ \phi_{m\;\mathrm{CA}} & ∼ & U\;[0.550,\;0.894]\\ {\left[\eta \right]}_{\mathrm{fa}} & ∼ & U\;[2.5,\;6.8]\\ {\left[\eta \right]}_{\mathrm{FA}} & ∼ & U\;[2.5,\;9.0]\\ {\left[\eta \right]}_{\mathrm{CA}} & ∼ & U\;[2.5,\;9.0]\\ \delta & ∼ & U\;[0,\;40]\\ \sigma & ∼ & U\;[0,\;400] \end{array}$$

**Figure 2:**
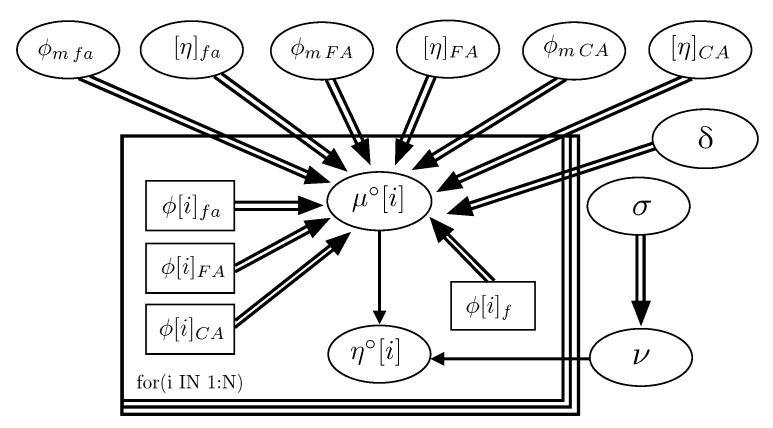
Bayesian network graph of the De La Rosa et al. model [15] for SCSFRC (from the investigation of Grünewald [32]).

**Figure 3:**
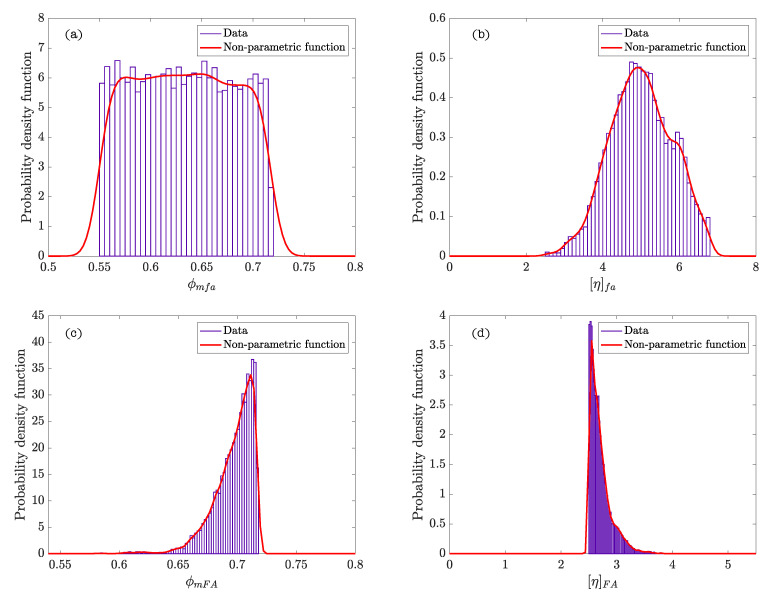
Probability density functions of $\phi_{m}$ and [η] for the powder phase ((**a**,**b**), respectively), and fine granular phase ((**c**,**d**), respectively) in SCSFRC (λ = 78.5).

**Figure 4:**
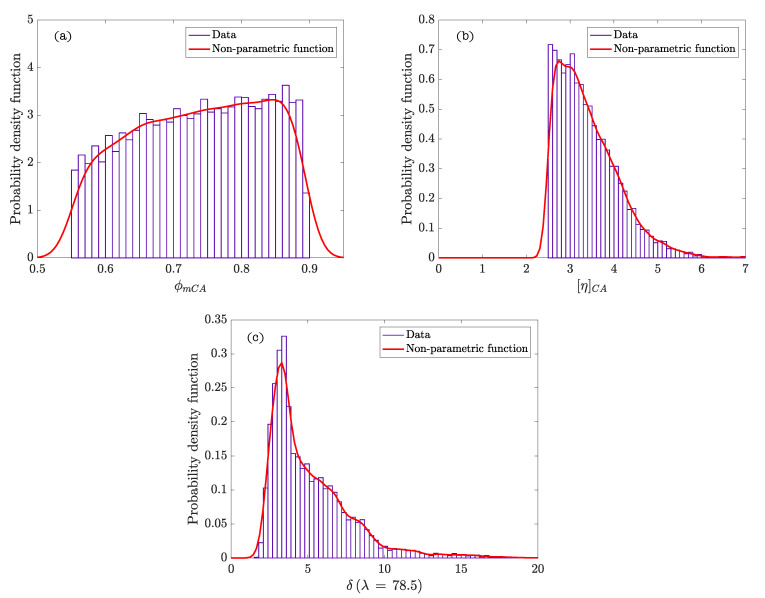
Probability density functions of $\phi_{m}$ and [η] for the coarse granular phase ((**a**,**b**), respectively) and δ parameter (**c**) in SCSFRC (λ = 78.5).

**Figure 5:**
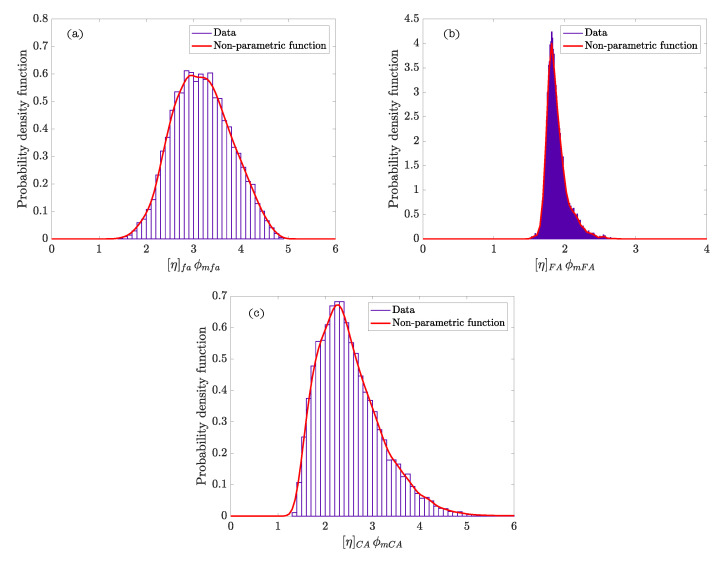
Probability density functions of $\phi_{m\;i}\;{\left[\eta \right]}_{i}$ ((**a**) powder phase; (**b**) fine granular phase; (**c**) coarse granular phase) in SCSFRC (λ = 78.5).

**Figure 6:**
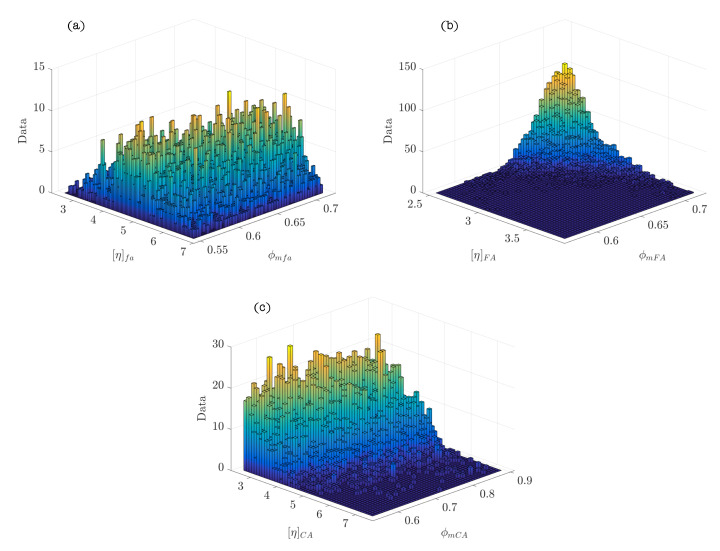
Bivariate histogram of $\phi_{m\;i}$ and ${\left[\eta \right]}_{i}$ ((**a**) powder phase; (**b**) fine granular phase; (**c**) coarse granular phase) composing SCSFRC (λ = 78.5).

Figure 2 represents the hierarchy and dependence structure of the variables of the Bayesian network of the model. Five different SCSFRCs have been studied, corresponding to five aspect ratios, (λ = 46.3, 64.3, 64.9, 78.5, and 85.7). Table 4 includes the statistics values obtained after the Bayesian analysis. Figure 3 and Figure 4 represent the probability density functions of the parameters for the phases of SCSFRC with λ = 78.5. The probability density function of the exponent of the Krieger and Dougherty equation ($\phi_{m\;i}\;{\left[\eta \right]}_{i}$) for the phases of SCSFRC is plotted in Figure 5. Finally, the bivariate histogram of the parameters $\phi_{m\;i}$ and ${\left[\eta \right]}_{i}$ of the phases for the SCSFRC with λ = 78.5 is shown in Figure 6.

In this case, the Bayesian analysis of the SCSFRC was done with three phases of the Krieger and Dougherty equation (one powder phase plus two granular phases), and one steel-fiber phase to verify the model of De La Rosa et al. [15]. The powder phase shows similar values of $\phi_{m\;\mathrm{fa}}$ for all the SCSFRC (≈0.63). However, the values of ${\left[\eta \right]}_{\mathrm{fa}}$ are more dispersed. If we observe the non-parametric density functions (Figure 3a), $\phi_{m\;\mathrm{fa}}$ shows a uniform density function in the same range of values, which is similar in the rest of the SCSFRCs not represented (λ = 46.3, 64.3, 64.9, and 85.7). However, ${\left[\eta \right]}_{\mathrm{fa}}$ (Figure 3b) shows a probability density function with a peak which grows as the aspect ratio of the fiber increases.

Regarding $\phi_{m\;\mathrm{FA}}$, the mean value is roughly 0.67; the same conclusion can be obtained with ${\left[\eta \right]}_{\mathrm{FA}}$ (≈3.0). Both non-parametric density functions (Figure 3c) show the same trend probability peaks about the same values in every SCSFRC.

The mean values obtained for $\phi_{m\;\mathrm{CA}}$ are 0.73 regardless of the specific type of SCSFRC. The mean values calculated for ${\left[\eta \right]}_{\mathrm{CA}}$ are approximately 5.0 except for those SCSFRC with a higher aspect ratio of the fiber, which figures lower than 5.0. The non-parametric density functions are approximately uniform for $\phi_{m\;\mathrm{CA}}$ (Figure 4a) in every SCSFRC. As to ${\left[\eta \right]}_{\mathrm{CA}}$, the density function shows a peak of different values depending on λ: The lower the aspect ratio, the higher the value ${\left[\eta \right]}_{\mathrm{CA}}$ with a maximum probability (Figure 4b).

Comparing these results with those obtained for the analysis of SCC with respect to the granular phases (fine and coarse aggregate), we realize that the values of the parameters are very similar. This fact means that the Bayesian analysis in two and three phases for the SCC offers approximately the same results and conclusions.

According to the results of the Bayesian analysis (Table 4), it is verified that the parameter δ can acquire values much higher than 3, except for the fiber with λ = 78.5 (δ≈ 5). This trend is the same as that previously observed while fixing the ranges of δ (Table 3). In Figure 4c we can observe the non-parametric density function of δ for a steel fiber with λ = 78.5, which reaches a clear peak of probability. For the rest of the λ values, this peak is not so clear, and the density functions are smoother but reach much higher values than those for λ = 78.5. Probably, this is because Equation (13) is an approximation of the contribution of the fiber in the effective stress tensor obtained by following the procedure of Phan–Thien and Karihaloo [29], who derive the effective stress tensor, and the fiber contributed stress from the slender body theory of Russel [29,52].

Figure 5 shows that the most probable value for the exponent of the Krieger and Dougherty equation is different from the theoretical value of 1.9. This is true in all phases except for the fine aggregate phase. The extension of the probability density function of the powder and coarse aggregate phases represents the range that the exponent could acquire. Finally, the bivariate histogram of the parameters $\phi_{m\;i}$ and ${\left[\eta \right]}_{i}$ of the phases of SCSFRC (λ = 78.5) is represented in Figure 6.

### 4.2. Application of the Bayesian Analysis Results to the Experimental Data

The same process used for self-compacting mortar and self-compacting concrete [21] was followed for SCSFRC. For this, the mean values of the parameters obtained in the Bayesian analysis of the model to the data of Grünewald (Table 5 and Table 6) [32] were applied. After, the results obtained were compared with those calculated using the values of Abo-Daheer et al. ($\phi_{m\;fa}$ = 0.524 for powder phase; $\phi_{m\;FA}$ = 0.63 for powder and fine aggregate phase; $\phi_{m\;CA}$ = 0.74 for powder, fine and coarse aggregate phases; $\phi_{m,i}\;{\left[\eta \right]}_{i}$ = 1.9) [14] and Ghanbari et al. (δ = 3) [22].

If we set an error of ≤25% between the experimental rheological measurements of Grünewald [32], and the estimation made with the mean of the parameters calculated with the Bayesian method, we obtain an excellent approximation of 80% of the global data. However, if we set the typical parameters used in the Krieger and Dougherty model proposed by Abo-Daheer et al. [14] for the terms of the model of De La Rosa et al. (Equation (12)) [15], and the parameter proposed by Ghanbari et al. [22] for the inclusion of steel fiber in the mentioned model [15], we obtain an approximation of 11% of the global data. Indeed, we obtain a good approximation of 70% of the global data if we use the simplest model (Equation (17)).

## 5. Conclusions

This article extends the research on the transformation of deterministic models into probabilistic models for the study of the dynamic viscosity in cementitious suspensions, in this case applying the methodology to self-compacting steel fiber reinforced concrete (SCSFRC). If the uncertainty associated with the nature, geometry and particle size distribution of cementitious suspensions already required considering the Krieger and Dougherty equation with random variables (in terms of its parameters), the inclusion of steel fibers in the system also advises using the Bayesian approach.

The Bayesian analysis was applied to a deterministic micromechanical model, which calculates the dynamic viscosity of SCSFRC, to obtain the samples of the variables as probability functions (density or distribution), which are the parameters of the deterministic models. Through the open-source software OpenBUGS, which employs Markov Chain Monte Carlo and Gibbs Sampling methods, the simulations were performed. An acyclic graph describes the hierarchy and independence of variables and conditions the probability density function of the parameters of the micromechanical model. The analysis attributes the calculated distributions to all the causes that physically condition them, not just to a single cause. The main results reached in this article are:

- The Bayesian methodology responds to questions in complex systems (fluid paste, aggregates and rigid fibers) with complex models (Krieger and Dougherty equation and De La Rosa et al. equation) about the probability of any parameter of those to reach a specific value in the function of the type of material employed.
- This change of paradigm about the use of probabilistic models in this type of systems can be useful for cementitious material designers, as well as for other engineering models.
- When the values of the parameters calculated through the Bayesian analysis are applied in the model, the approximation to the experimentally measured values of dynamic viscosity in SCSFRC is better than the theoretical values suggested by the scientific literature (calculations using the Bayesian mean values were better than those made with the theoretical values, considerably decreasing the error).

These results indicate the usefulness of Bayesian analysis in obtaining better estimates of the models used in engineering and science.

## Figures and Tables

**Table 1:** Composition of base SCC [32] (w: Water; SP LR and HR: Superplasticizers; fa: Fly ash; FA: Fine aggregate, CA: Coarse aggregate).

	CEM I 52.5 R	CEM III 42.5 N	w	SP LR + SP HR	fa	FA	CA	$\eta_{\mathrm{SCC}}$	$\eta_{p}$
Denomination	[kg/m${}^{3}$]	[kg/m${}^{3}$]	[kg/m${}^{3}$]	[kg/m${}^{3}$]	[kg/m${}^{3}$]	[kg/m${}^{3}$]	[kg/m${}^{3}$]	[Pa s]	[Pa s]
OS1	249	155	172	2.58 + 1.58	142	913	682	69.2	0.404
OS2	263	149	181	2.88 + 1.44	173	876	655	59.4	0.413
OS3	249	149	171	2.59 + 2.12	146	1089	508	87.9	0.413
OS4	269	143	181	2.78 + 1.85	173	1045	487	56.0	0.413
OS5	0	335	155	2.10 + 1.26	168	1134	528	97.6	0.413
OS6	0	352	164	2.10 + 1.18	192	1089	508	81.0	0.422
OS7	0	367	173	2.17 + 1.09	217	1045	487	62.2	0.422
OS8	228	151	181	2.68 + 1.49	166	1100	467	71.3	0.395
OS9	246	164	188	2.73 + 1.31	180	1058	449	57.5	0.404

**Table 2:** λ, $\phi_{f}$ and $\eta_{SCSFRC}$ of SCSFRC. Test set-up of measurements with BML–Viscometer [32].

Denomination	λ	$\phi_{f}$	$\eta_{\mathrm{SCSFRC}}$ [Pa s]	Denomination	λ	$\phi_{f}$	$\eta_{\mathrm{SCSFRC}}$ [Pa s]
OS1 80/30	78.5	0.008	167.8	OS5 80/30	78.5	0.005	195.8
OS1 80/60 BP	85.7	0.005	122.9	OS5 80/30	78.5	0.008	326.2
OS1 80/60 BP	85.7	0.008	125.0	OS5 80/60 BP	85.7	0.005	187.2
OS1 45/30	46.3	0.013	137.5	OS5 80/60 BP	85.7	0.008	261.8
OS1 80/30	78.5	0.005	116.8	OS5 45/30	46.3	0.013	245.3
OS1 45/30	46.3	0.010	109.9	OS5 45/30	46.3	0.015	280.3
OS2 80/30	78.5	0.008	171.1	OS6 80/30	78.5	0.008	266.8
OS2 80/30	78.5	0.010	223.2	OS6 80/30	78.5	0.010	344.2
OS2 80/60 BP	85.7	0.005	98.6	OS6 80/60 BP	85.7	0.005	182.8
OS2 80/60 BP	85.7	0.008	159.9	OS6 80/60 BP	85.7	0.008	301.8
OS2 45/30	46.3	0.018	262.0	OS6 45/30	46.3	0.015	211.5
OS2 45/30	46.3	0.015	144.3	OS6 45/30	46.3	0.018	265.0
OS3 80/30	78.5	0.005	143.1	OS7 80/30	78.5	0.008	209.1
OS3 80/30	78.5	0.008	199.3	OS7 80/30	78.5	0.010	306.1
OS3 80/60 BP	85.7	0.005	124.3	OS7 80/60 BP	85.7	0.008	224.8
OS3 80/60 BP	85.7	0.008	154.8	OS7 80/60 BP	85.7	0.010	233.1
OS3 45/30	46.3	0.015	237.0	OS7 65/40	64.9	0.013	206.1
OS3 45/30	46.3	0.018	279.3	OS7 45/30	46.3	0.015	157.1
OS4 80/30	78.5	0.010	245.3	OS7 45/30	46.3	0.018	204.4
OS4 80/60 BP	85.7	0.008	102.3	OS7 65/40	64.9	0.010	155.2
OS4 80/60 BP	85.7	0.010	199.7	OS8 80/30	78.5	0.003	80.8
OS4 45/30	46.3	0.018	145.7	OS8 80/30	78.5	0.005	141.4
OS4 65/40	64.9	0.015	221.1	OS8 65/20	64.3	0.005	98.8
OS4 80/30	78.5	0.008	156.5	OS8 65/20	64.3	0.008	210.1
OS4 45/30	46.3	0.015	117.5	OS9 80/30	78.5	0.005	92.2
OS4 45/30	46.3	0.020	176.1	OS9 80/30	78.5	0.008	162.4
OS4 65/40	64.9	0.013	182.2	OS9 65/20	64.3	0.005	120.7
				OS9 65/20	64.3	0.008	177.3
				OS9 65/20	64.3	0.010	142.6

**Table 3:** Statistics of the parameter δ obtained for different aspect ratio values.

Aspect Ratio (λ)	Parameter	Mean	Std. Dev.	Percentage 2.5%	Median	Percentage 97.5%
46.3		14.350	1.819	11.570	14.100	18.640
64.3		16.950	6.000	9.954	15.170	34.610
64.9	δ	16.000	2.974	12.890	15.400	24.510
78.5		14.860	1.227	12.720	14.750	17.610
85.7		22.560	3.261	17.680	22.050	30.660

**Table 4:** Statistics values of the parameters $\phi_{m\;i}$, ${\left[\eta \right]}_{i}$, and $\delta_{i}$ for SCSFRC.

Aspect Ratio (λ)	Parameter	Mean	Std. Dev.	Percentage 2.5%	Median	Percentage 97.5%
	$\phi_{m\;\mathrm{fa}}$	0.637	0.048	0.555	0.638	0.713
	${\left[\eta \right]}_{\mathrm{fa}}$	3.811	0.954	2.558	3.636	6.120
	$\phi_{m\;\mathrm{FA}}$	0.671	0.031	0.604	0.675	0.715
46.3	${\left[\eta \right]}_{\mathrm{FA}}$	3.131	0.481	2.525	3.028	4.273
	$\phi_{m\;\mathrm{CA}}$	0.726	0.099	0.560	0.729	0.886
	${\left[\eta \right]}_{\mathrm{CA}}$	5.237	1.241	2.863	5.207	7.672
	δ	14.160	9.892	2.638	11.260	37.380
	$\phi_{m\;\mathrm{fa}}$	0.633	0.048	0.554	0.633	0.713
	${\left[\eta \right]}_{\mathrm{fa}}$	4.603	1.221	2.612	4.572	6.680
	$\phi_{m\;\mathrm{FA}}$	0.663	0.036	0.586	0.669	0.715
64.3	${\left[\eta \right]}_{\mathrm{FA}}$	3.306	0.507	2.545	3.258	4.409
	$\phi_{m\;\mathrm{CA}}$	0.723	0.099	0.559	0.722	0.885
	${\left[\eta \right]}_{\mathrm{CA}}$	5.424	1.785	2.649	5.338	8.723
	δ	20.110	10.290	4.039	19.350	38.730
	$\phi_{m\;\mathrm{fa}}$	0.638	0.048	0.555	0.641	0.714
	${\left[\eta \right]}_{\mathrm{fa}}$	3.509	0.923	2.527	3.218	5.966
	$\phi_{m\;\mathrm{FA}}$	0.650	0.045	0.562	0.656	0.714
64.9	${\left[\eta \right]}_{\mathrm{FA}}$	3.268	0.541	2.534	3.174	4.495
	$\phi_{m\;\mathrm{CA}}$	0.726	0.100	0.559	0.730	0.886
	${\left[\eta \right]}_{\mathrm{CA}}$	5.333	1.806	2.624	5.113	8.689
	δ	20.230	10.200	4.677	19.350	38.740
	$\phi_{m\;\mathrm{fa}}$	0.633	0.048	0.554	0.633	0.713
	${\left[\eta \right]}_{\mathrm{fa}}$	5.007	0.810	3.419	4.987	6.531
	$\phi_{m\;\mathrm{FA}}$	0.697	0.017	0.657	0.701	0.716
78.5	${\left[\eta \right]}_{\mathrm{FA}}$	2.706	0.195	2.506	2.652	3.223
	$\phi_{m\;\mathrm{CA}}$	0.736	0.096	0.563	0.742	0.887
	${\left[\eta \right]}_{\mathrm{CA}}$	3.407	0.696	2.533	3.261	5.104
	δ	5.184	2.765	2.318	4.357	12.440
	$\phi_{m\;\mathrm{fa}}$	0.623	0.048	0.553	0.618	0.711
	${\left[\eta \right]}_{\mathrm{fa}}$	5.928	0.731	3.974	6.105	6.770
	$\phi_{m\;\mathrm{FA}}$	0.675	0.027	0.620	0.678	0.715
85.7	${\left[\eta \right]}_{\mathrm{FA}}$	3.042	0.352	2.527	3.002	3.815
	$\phi_{m\;\mathrm{CA}}$	0.733	0.098	0.562	0.737	0.887
	${\left[\eta \right]}_{\mathrm{CA}}$	4.002	0.871	2.615	3.936	5.860
	δ	20.480	8.325	7.849	19.180	38.060

**Table 5:** Experimental values, models’ values and estimated error for SCSFRC (Grünewald [32]: Series OS1–OS4).

Denomination	Experimental	Bayesian Calculus	Bayesian Calculus	Theoretical Calculus	Error with Bayesian	Error with Bayesian	Error with Theoretical
	$\eta_{\mathrm{SCSFRC}}$ [Pa s]	Equation (17), $\eta_{\mathrm{SCSFRC}}$ [Pa s]	Equation (12), $\eta_{\mathrm{SCSFRC}}$ [Pa s]	[14,22], $\eta_{\mathrm{SCSFRC}}$ [Pa s]	Calculus Equation (17) [%]	Calculus Equation (12) [%]	Calculus [14,22] [%]
OS1 80/30	167.8	205.5	161.7	270.8	22.5	3.6	61.4
OS1 80/60 BP	122.9	139.3	108.8	217.1	13.4	11.5	76.6
OS1 80/60 BP	125.0	174.4	137.5	313.0	39.5	10.0	150.4
OS1 45/30	137.5	160.6	132.2	184.0	16.8	3.8	33.8
OS1 80/30	116.8	160.1	115.9	189.0	37.0	0.8	61.8
OS1 45/30	109.9	142.3	117.1	152.3	29.5	6.5	38.6
OS2 80/30	171.1	176.4	139.4	206.8	3.1	18.6	20.8
OS2 80/30	223.2	215.4	178.8	269.3	3.5	19.9	20.6
OS2 80/60 BP	98.6	119.6	91.3	165.7	21.3	7.4	68.1
OS2 80/60 BP	159.9	149.7	115.4	239.0	6.4	27.8	49.4
OS2 45/30	262.0	169.2	122.9	189.0	35.4	53.1	27.9
OS2 45/30	144.3	153.5	111.5	164.7	6.4	22.8	14.2
OS3 80/30	143.1	203.3	133.8	306.6	42.1	6.5	114.2
OS3 80/30	199.3	261.0	186.7	439.4	31.0	6.3	120.5
OS3 80/60 BP	124.3	177.0	132.4	352.2	42.4	6.6	183.3
OS3 80/60 BP	154.8	221.5	167.4	507.8	43.1	8.1	228.1
OS3 45/30	237.0	227.2	160.0	350.1	4.2	32.5	47.7
OS3 45/30	279.3	250.4	176.5	401.7	10.4	36.8	43.8
OS4 80/30	245.3	203.3	190.4	373.6	17.2	22.4	52.3
OS4 80/60 BP	102.3	141.1	126.2	331.5	37.9	23.3	224.1
OS4 80/60 BP	199.7	169.5	152.5	433.1	15.1	23.6	116.9
OS4 45/30	145.7	159.5	121.8	262.2	9.5	16.4	80.0
OS4 65/40	221.1	201.5	159.2	396.1	8.9	28.0	79.2
OS4 80/30	156.5	166.3	148.4	286.9	6.3	5.2	83.3
OS4 45/30	117.5	144.7	110.4	228.6	23.2	6.0	94.5
OS4 45/30	176.1	174.3	133.1	295.9	1.0	24.4	68.0
OS4 65/40	182.4	177.2	141.3	334.6	2.8	22.5	83.4

**Table 6:** Experimental values, models’ values, and estimated error for SCSFRC (Grünewald [32]): Series OS5–OS9).

Denomination	Experimental	Bayesian Calculus	Bayesian Calculus	Theoretical Calculus	Error with Bayesian	Error with Bayesian	Error with Theoretical
	$\eta_{\mathrm{SCSFRC}}$ [Pa s]	Equation (17), $\eta_{\mathrm{SCSFRC}}$ [Pa s]	Equation (12), $\eta_{\mathrm{SCSFRC}}$ [Pa s]	[14,22], $\eta_{\mathrm{SCSFRC}}$ [Pa s]	Calculus Equation (17) [%]	Calculus Equation (12) [%]	Calculus [14,22] [%]
OS5 80/30	195.8	225.7	236.3	373.6	15.3	20.7	241.0
OS5 80/30	326.2	289.8	329.7	331.5	11.2	1.1	193.4
OS5 80/60 BP	187.2	196.5	273.3	433.1	5.0	46.0	309.8
OS5 80/60 BP	261.8	245.9	345.4	262.2	6.1	31.9	322.5
OS5 45/30	245.3	226.5	271.6	396.1	7.7	10.7	165.1
OS5 45/30	280.3	252.2	302.6	396.1	10.0	8.0	172.1
OS6 80/30	266.8	240.5	242.5	525.9	9.9	9.1	97.1
OS6 80/30	344.2	293.7	311.2	684.9	14.7	9.6	99.0
OS6 80/60 BP	182.8	163.1	185.4	421.5	10.8	1.4	130.6
OS6 80/60 BP	301.8	204.1	234.3	607.8	32.4	22.4	101.4
OS6 45/30	211.5	209.3	188.6	419.0	1.0	10.8	98.1
OS6 45/30	265.0	230.7	208.0	480.7	12.9	21.5	81.4
OS7 80/30	209.1	184.7	195.8	353.4	11.7	6.4	69.0
OS7 80/30	306.1	225.5	251.2	460.2	26.3	17.9	50.3
OS7 80/60 BP	224.8	156.7	179.0	408.4	30.3	20.4	81.7
OS7 80/60 BP	233.1	188.2	216.3	533.6	19.2	7.2	128.9
OS7 65/40	206.1	196.9	166.9	412.1	4.5	19.0	100.0
OS7 45/30	157.1	160.7	133.3	281.6	2.3	15.1	79.2
OS7 45/30	204.4	177.2	147.0	323.0	13.3	28.1	58.0
OS7 65/40	155.2	169.9	145.8	336.3	9.5	6.0	116.7
OS8 80/30	80.8	118.1	67.8	138.4	46.2	16.1	71.3
OS8 80/30	141.4	164.9	112.1	244.3	16.6	20.7	72.7
OS8 65/20	98.8	128.6	124.2	180.4	30.2	25.7	82.6
OS8 65/20	210.1	157.3	149.3	254.3	25.1	28.9	21.0
OS9 80/30	92.2	133.0	93.0	174.6	44.2	0.9	89.3
OS9 80/30	162.4	170.7	129.7	250.2	5.1	20.1	54.1
OS9 65/20	120.7	103.7	90.5	128.9	14.1	25.0	6.8
OS9 65/20	177.3	126.8	108.8	181.7	28.5	38.6	2.5
OS9 65/20	142.6	150.0	127.1	234.6	5.2	10.9	64.5

## Data Availability

Not applicable.

## Abbreviations

SCC	Self-compacting concrete
SCSFRC	Self-compacting steel-fiber reinforced concrete
*i*	Number of nodes
*j*	Number of phase of SCC
*n*	Number of conditional probability density functions
*N*	Normal probability density function
*U*	Uniform probability density function
$\varepsilon^{\circ }$	Residual error for non-dimensional dynamic viscosity of SCC
δ	Parameter of the system when adding the steel fiber
η	Dynamic viscosity
$\eta_{\mathrm{SCC}}$	Dynamic viscosity of SCC
$\eta_{p}$	Cement paste dynamic viscosity
$\eta_{0}$	Fluid phase dynamic viscosity
$\eta^{\circ }$	Non–dimensional dynamic viscosity of SCC
$\eta^{⋄}$	=$\frac{\eta_{\mathrm{SCSFRC}}}{\eta_{SCC}}$: non-dimensional viscosity of SCSFRC
[η]	Intrinsic viscosity
${\left[\eta \right]}_{\mathrm{CA}}$	Intrinsic viscosity of the coarse aggregate phase in SCC
${\left[\eta \right]}_{\mathrm{fa}}$	Intrinsic viscosity of the powder phase in SCC
${\left[\eta \right]}_{\mathrm{FA}}$	Intrinsic viscosity of the fine aggregate phase in SCC
λ	Aspect ratio of steel fiber
$\mu^{\circ }$	Mean value for non-dimensional dynamic viscosity of SCC
$\mu^{⋄}$	Mean value for non-dimensional dynamic viscosity of SCSFRC
$\nu =\frac{1}{\sigma^{2}}$	Auxiliar variable for the model of probability
$\pi_{i}$	Set of nodes $X_{i}$ in G
σ	Standard deviation of the sample
$\phi_{f}$	Volume fraction of steel fiber
$\phi_{\mathrm{CA}}$	Coarse aggregate volume fraction
$\phi_{\mathrm{fa}}$	Powder volume fraction
$\phi_{\mathrm{FA}}$	Fine aggregate volume fraction
$\phi_{m}$	Maximum packing density of particles
$\phi_{m\;\mathrm{CA}}$	Maximum packing density of particles in the coarse aggregate phase in SCSFRC
$\phi_{m\;\mathrm{fa}}$	Maximum packing density of particles in the powder phase in SCSFRC
$\phi_{m\;\mathrm{FA}}$	Maximum packing density of particles in the fine aggregate phase in SCSFRC
$\phi_{\lambda }$	Function which depends on the π number and the aspect ratio of the steel fiber
